# The Impact of COVID-19 on DTP3 Vaccination Coverage in Europe (2012–2023)

**DOI:** 10.3390/vaccines13010006

**Published:** 2024-12-24

**Authors:** Ines Aguinaga-Ontoso, Sara Guillen-Aguinaga, Laura Guillen-Aguinaga, Rosa Alas-Brun, Miriam Guillen-Aguinaga, Luc Onambele, Enrique Aguinaga-Ontoso, Esperanza Rayón-Valpuesta, Francisco Guillen-Grima

**Affiliations:** 1 Department of Health Sciences, Public University of Navarra, 31008 Pamplona, Spain; ines.aguinaga@unavarra.es (I.A.-O.);; 2 Healthcare Research Institute of Navarra (IdiSNA), 31008 Pamplona, Spain; 3 CIBER in Epidemiology and Public Health (CIBERESP), Institute of Health Carlos III, 46980 Madrid, Spain; 4 San Juan Primary Health Care Center, Navarra Health Service, 31008 Pamplona, Spain; 5 Department of Nursing, Kystad Helse-og Velferdssenter, 7026 Trondheim, Norway; 6 Neoma Business School, 75013 Paris, France; 7 School of Health Sciences, Catholic University of Central Africa, Yaoundé 1110, Cameroon; onambele.luc@ess-ucac.org; 8 Department of Sociosanitary Sciences, University of Murcia, 30003 Murcia, Spain; aguinaga@um.es; 9 Department of Preventive Medicine, Virgen de la Arrixaca University Clinical Hospital, 30003 Murcia, Spain; 10 Department of Nursing, Complutense University of Madrid, 28040 Madrid, Spain; erayon@ucm.es; 11 Department of Preventive Medicine, Clínica Universidad de Navarra, 31008 Pamplona, Spain

**Keywords:** DTP3 vaccination, COVID-19 pandemic impact, vaccination trends, Europe

## Abstract

Background: The COVID-19 pandemic disrupted routine child immunization efforts, threatening to reverse progress in controlling vaccine-preventable diseases. Materials and Methods: We analyzed the impact of COVID-19 on DTP3 vaccination in Europe by comparing trends before and after the pandemic using time series data from 2000 to 2023. Employing joinpoint regression, chi-square tests, and segmented regression analysis, we assessed DTP3 vaccination trends and coverage changes. Results: The findings revealed significant regional disparities across Europe. Statistical models indicated reductions in DTP3 coverage in countries such as Ireland, Sweden, and Switzerland, whereas Ukraine and San Marino showed improvements. Conclusions: There are variations in the effect of COVID-19 on DTP3 coverage rates, indicating the need for targeted public health strategies to address vaccine hesitancy, logistical barriers, and systemic inequities.

## 1. Introduction

Routine immunization is crucial for reducing childhood mortality and morbidity. Diphtheria, tetanus, and pertussis vaccination (DTP) is a cornerstone of childhood immunization programs and is essential for overall program performance and public health efficacy. Countries with high DTP3 coverage maintain herd immunity and reduce outbreak risks [1,2].

### 1.1. Impact of Pandemics on Immunization Programs

Historically, pandemics have disrupted immunization programs by diverting resources and limiting healthcare access [3]. During the COVID-19 pandemic, lockdowns, physical distancing, and reallocating healthcare resources led to declining child vaccination rates globally [4,5]. This decline contributed to an “immunity debt” as reduced exposure to pathogens increased population susceptibility, underscoring the need for consistent immunization to prevent a resurgence of vaccine-preventable diseases [6].

### 1.2. Declines in DTP3 Coverage During the COVID-19 Pandemic

The COVID-19 pandemic caused unprecedented disruptions to routine immunization programs worldwide [7,8,9,10,11,12]. The percentage of one-year-olds receiving the DTP3 vaccine dropped globally from 86% in 2019 to 81% in 2021, with a partial recovery to 84% by 2023. Prior research has shown significant declines in DTP3 vaccination coverage in Africa and the Americas due to the pandemic, highlighting healthcare access and delivery disparities [13,14].

### 1.3. Vaccine Hesitancy and the Role of Misinformation

The pandemic intensified vaccine hesitancy, partially driven by misinformation on social media platforms. Misinformation about COVID-19 may have influenced attitudes toward other vaccines, including those for children. This “infodemic” fostered doubt among parents, potentially affecting routine childhood vaccinations [8].

Concerns about contracting COVID-19 at healthcare facilities may have led to vaccine hesitancy among parents. In Europe, declining routine vaccination rates in children, including DTP3, have raised concerns [15]. In this sense, the WHO reported an alarming rise in European measles cases, likely linked to vaccination coverage disruptions [16].

### 1.4. DTP3 Vaccination in Europe

In Europe, which has DTP vaccination programs in 42 countries, high levels of DTP3 coverage have been maintained through robust healthcare infrastructures [17]. However, the pandemic disrupted this stability. The WHO reported a slight decrease in the European Region’s DTP3 coverage, dropping from 95% in 2019 to 94% in 2020. While this reduction seems minimal, it strains healthcare systems due to logistical challenges, healthcare worker shortages, and movement restrictions [15,16,18,19,20,21,22,23,24,25,26,27].

### 1.5. Challenges in Routine Vaccination Services

The COVID-19 pandemic presented unprecedented challenges to healthcare systems, disrupting routine vaccination services due to lockdowns, travel restrictions, and reallocating resources [28,29,30,31].

The pandemic may have disrupted routine DTP3 vaccination programs due to logistical challenges [15,26,27,32,33,34]. While some European countries may have recovered to pre-pandemic vaccination levels, others might still struggle due to persistent logistical issues or increased vaccine hesitancy [34,35]. We will compare DTP3 coverage in 2019 and 2023 to explore these potential changes, assessing whether vaccination rates have returned to pre-pandemic levels or remain affected.

### 1.6. Research Questions and Objectives

The research question guiding this study is: How did the COVID-19 pandemic affect DTP3 vaccination coverage in Europe, and have vaccination rates returned to pre-pandemic levels? This question explores whether the pandemic caused a temporary disruption or a lasting shift in routine immunization programs. Additionally, it seeks to uncover regional disparities in recovery, examining how logistical challenges, vaccine hesitancy, or other factors hinder the restoration of DTP3 coverage to pre-pandemic levels. By addressing this question, the study contributes to understanding the broader implications of the pandemic on European public health and immunization systems.

This study examines whether the COVID-19 pandemic caused an abrupt decline in DTP3 coverage in Europe and assesses if vaccination trends have returned to pre-pandemic levels. By analyzing data from 2012 to 2023, we aim to determine whether the pandemic merely interrupted existing trends or significantly shifted vaccination coverage trajectories. Understanding variations across different European regions can highlight disparities in recovery and identify areas where vaccine hesitancy or logistical challenges continue to hinder full recovery [35,36,37].

## 2. Materials and Methods

### 2.1. Data Source and Processing

Information on vaccination DTP3 rates for European countries was obtained from the United Nations Children’s Fund’s databases, covering 2012 to 2023 [38]. We consider European countries included in the United Nations geoscheme for Europe [39]. The analysis excluded several territories that, while geographically located within Europe, possess distinct political statuses not typically associated with independent sovereign nations (Åland Islands, Faroe Islands, Isle of Man, Svalbard and Jan Mayen Islands, Guernsey, Jersey, Sark, and Gibraltar). These territories often function as dependencies, crown dependencies, or overseas territories of other European nations, exhibiting varying degrees of autonomy and self-governance. We also excluded the Vatican, the Republic of Kosovo, and Liechtenstein because no datum was available. Regional data were also obtained from the UNICEF [38] repository.

The UNICEF database contains the annual rate of DTP3 vaccination per year and country. This database was used directly for the jointpoint regression and segmented regression analyses. Using UNICEF vaccination rates and considering the number of live births in each country, a database with the number of DTP3 vaccinated per year was elaborated. The database with the number of people vaccinated was used to calculate the chi-square test.

### 2.2. European Regions Analysis

Consolidated data for Europe and West Europe were sourced from UNICEF. Because there were no regional data from North and South Europe in UNICEF, we computed it by weighting national countries’ DTP3 vaccination rates with the number of births. Annual newborn data were collected per country from the World Bank, United Nations, and UNICEF [40,41,42].

The analysis in this study was divided into four European regions. Eastern Europe encompasses Belarus, Bulgaria, Czechia, Hungary, Poland, Moldova, Romania, Slovakia, and Ukraine. The Russian Federation was also included in this group for statistical convenience, although it is a transcontinental country. The same happened with Türkiye. Northern Europe includes Estonia, Ireland, Finland, Sweden, the United Kingdom, Latvia, Iceland, Lithuania, Norway, and Denmark. Southern Europe contains Albania, Andorra, Bosnia and Herzegovina, Cyprus, Croatia, Greece, Italy, Malta, Montenegro, North Macedonia, Portugal, San Marino, Serbia, Slovenia, and Spain. Lastly, Western Europe includes Austria, Belgium, France, Germany, Luxembourg, Monaco, the Netherlands, and Switzerland.

### 2.3. Statistical Analysis

We used various statistical techniques to evaluate how the COVID-19 pandemic affected European vaccination trends. First, we used a Joinpoint regression (piecewise linear regression) to detect changes in DTP3 vaccination. After that, we computed a chi-square test to compare vaccination coverage in 2019 (pre-pandemic) and 2023 (post-pandemic), examining whether the pandemic caused significant shifts in vaccinated versus unvaccinated proportions. Additionally, we conducted a segmented regression analysis covering the period from 2000 to 2023 to detect changes in long-term vaccination trends, mainly focusing on potential interruptions caused by the pandemic.

#### 2.3.1. Joinpoint Regression

Joinpoint regression is a statistical method used to identify points where significant changes in data trends occur. Joinpoint regression was utilized to find statistically significant results. Joinpoints, where abrupt changes in vaccination trends occurred, allowed us to pinpoint key moments linked to the pandemic’s impact.

The annual percentage change (APC) was computed using JoinPoint regression to quantify the magnitude of variation in DTP3 vaccination trends over time [43]. The Joinpoint regression models assumed constant variance.

This technique assumes that the data can be segmented into continuous linear pieces, with each segment representing a distinct trend. A fundamental assumption of JoinPoint regression is linearity within each segment, meaning that the relationship between the independent and dependent variables is linear within the identified intervals. Additionally, the model assumes continuity at the joinpoints, ensuring no abrupt jumps between segments, which is critical for the validity of the regression. Another important assumption is homoscedasticity, the constancy of residual variance across all segments. This ensures that the spread of residuals is consistent throughout the data [43].

The independence of residuals is also a key assumption, particularly relevant for time series data, where autocorrelation among residuals can bias results [44]. A Durbin–Watson test was computed to check for autocorrelation in the time series data [45]. If first-order autocorrelation was detected, it was accounted for in the analysis.

Joinpoints represent significant changes in trends, and their associated 95% confidence intervals (CIs) provide a range of plausible years for these changes based on the statistical model. To evaluate the association of joinpoints with the COVID-19 pandemic, we considered joinpoints to be “close to the pandemic” if the years 2020 and 2021 were within the 95% CI. This criterion reflects the flexibility required in interpreting the timing of trend changes when CIs encompass 2020 and 2021. These methods offer a comprehensive approach, combining hypothesis-driven and exploratory analyses to quantify the pandemic’s effect on vaccination coverage. Based on the data in Tables S1 and S2.

To complement the JoinPoint analysis, we compared each country’s DTP3 vaccination coverage between 2019 (the last pre-pandemic year) and 2023 (the most recent year in our dataset). Chi-square tests were employed to assess the statistical significance of any observed differences in coverage rates.

#### 2.3.2. Segmented Regression

We employed segmented regression analysis to assess how the COVID-19 pandemic impacted DTP3 vaccination trends in Europe from 2000 to 2023. This methodology, also known as interrupted time series analysis, is a robust quasi-experimental approach well-suited for evaluating the effects of external events or interventions on time series data. Our study period has spanned 23 years, with 20 years pre-pandemic (2000–2019) and three years’ post-pandemic (2020–2023).

The segmented regression model was defined as follows:

DTP3*t* = Intercept + *β*_1_Year + *β*_2_Pandemic + *β*_3_Interaction + *εt*

where:

- “DTP3*t*” represents the DTP3 vaccination coverage in year *t*.
- Year is the year of the calendar.
- “Pandemic” is a dummy variable, taking 1 for the COVID-19 pandemic years (2020–2023) and 0 for pre-pandemic years.
- Interaction is the interaction between Year and Pandemic.

If autocorrelation was detected with the Durbin–Watson test [45], the segmented regression model was adjusted by incorporating a lagged variable of the year to account for the observed serial dependence [46].

The model allows us to estimate the change in the level (immediate effect) of DTP3 vaccination coverage following the onset of the COVID-19 pandemic. Following the initial models that included year and pandemic as independent variables, we extended the analysis by testing models incorporating an interaction term between year and pandemic to assess whether the effect of time on DTP3 vaccination coverage varied between the pre-pandemic and pandemic periods. The interaction term allows us to capture how the relationship between year and vaccination coverage was modified during the pandemic; for example, the coverage of DTP3 was increasing before and after the pandemic began to decrease.

Segmented regression analysis relies on several key assumptions. First, it assumes that the relationship between time and the outcome variable, in this case, DTP3 vaccination coverage, is linear within each time series segment. This means that within both the pre-pandemic and pandemic periods, changes in vaccination coverage are adequately captured by distinct straight-line trends [47,48]. Additionally, the analysis presumes that the residuals, or errors, are independent across time points. Another important assumption is homoscedasticity, where the variance of the residuals is constant across all time points [49,50]. The model also assumes that the intervention point—the onset of the COVID-19 pandemic—is correctly specified as the point of change in the time series [51]. Finally, the analysis assumes the absence of unmeasured confounders that vary systematically over time and could influence vaccination coverage trends independently of the pandemic.

### 2.4. Software and Tools

All statistical analyses were conducted using specialized software and programming tools. Joinpoint regression was computed with Joinpoint Trend Analysis Software (Version 5.0.2, May 2023) [52,53]. Vaccination rates between 2019 and 2023 were compared using IBM SPSS Statistics (Version 29, IBM Corp., Armonk, NY, USA). The Durbin–Watson statistic was also computed with IBM SPSS. The segmented regression analysis, a form of interrupted time series analysis, was implemented in the R statistical environment (Version 4.3.1, 16 June 2023 ucrt) [54] utilizing the “segmented” package [54,55,56,57,58]. Data visualization was enhanced with the “ggplot2” package [59], and the “readxl” package facilitated the importation of data from Excel files [60]. We draw the map using the free software Mapchart (v 6.0.10) [61]. The color schemes for these maps were designed using ColorBrewer (Version 2.0) [62,63].

## 3. Results

**Figure 1:**
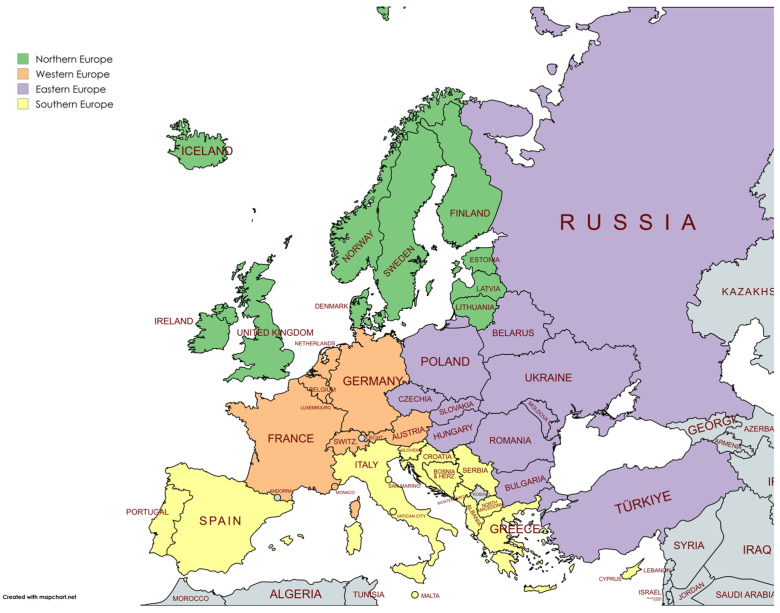
The United Nations Classification of European Regions.

**Figure 2:**
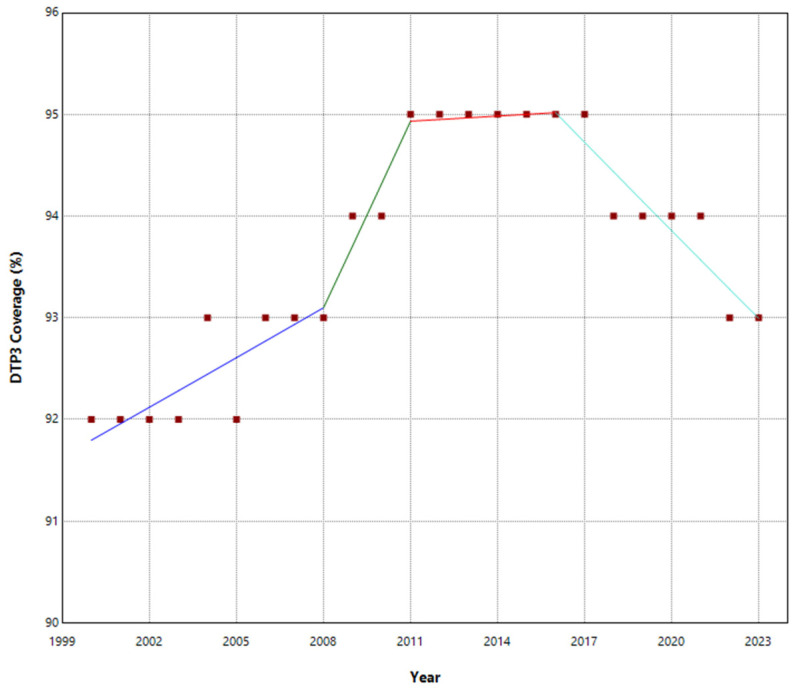
Joinpoint graph of DTP3 in North Europe, 2000–2022, indicates joinpoints at the transitions between colored lines.

**Figure 3:**
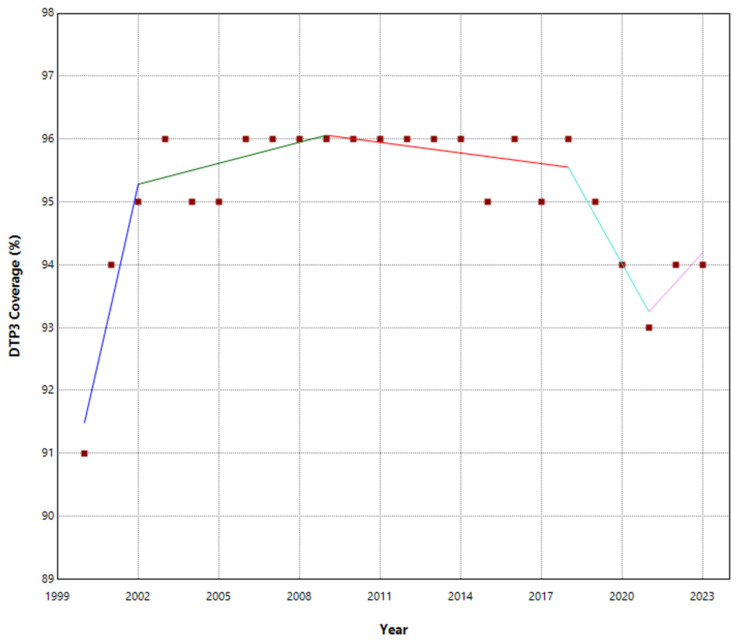
Joinpoint graph of DTP3 in South Europe, 2000–2022, indicates joinpoints at the transitions between colored lines.

**Figure 4:**
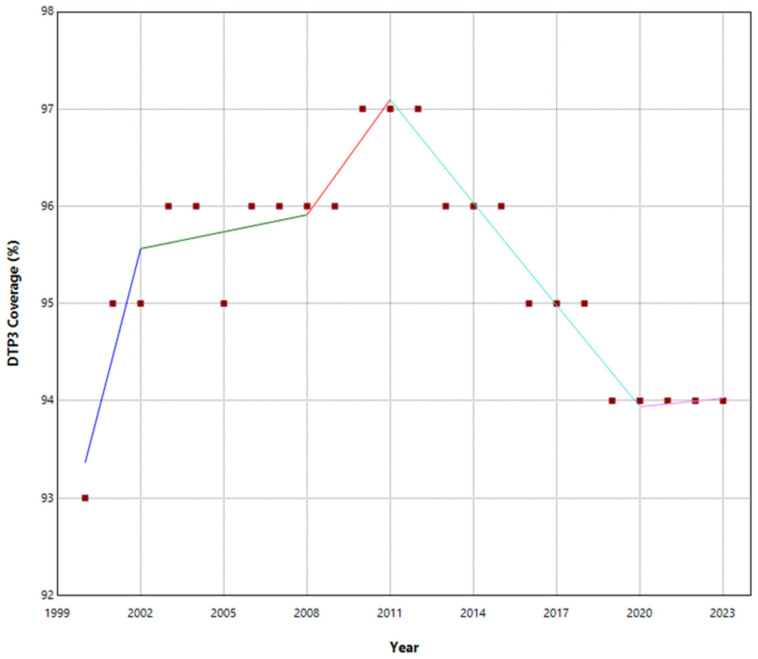
Joinpoint graph of DTP3 in West Europe, 2000–2022, indicates joinpoints at the transitions between colored lines.

No joinpoints were explicitly identified in Europe at the beginning of the COVID-19 pandemic (Table 1, Figure 1). There were joinpoints during the pandemic in South and West Europe (Table S1). Europe exhibited no significant trend changes overall, maintaining a stationary annual percent change (APC) of 0.03%. In contrast, Northern Europe experienced three joinpoints (Figure 2). Southern and Western Europe had four joinpoints (Figure 3 and Figure 4). East Europe had an upward trend with no joinpoint and an APC of 0.11% (Table 1). In Southern Europe and Western Europe before the COVID-19 pandemic, the DTP3 coverage had a negative trend. After the pandemic, coverage increased in Southern Europe and remained static in Western Europe.

**Figure 5:**
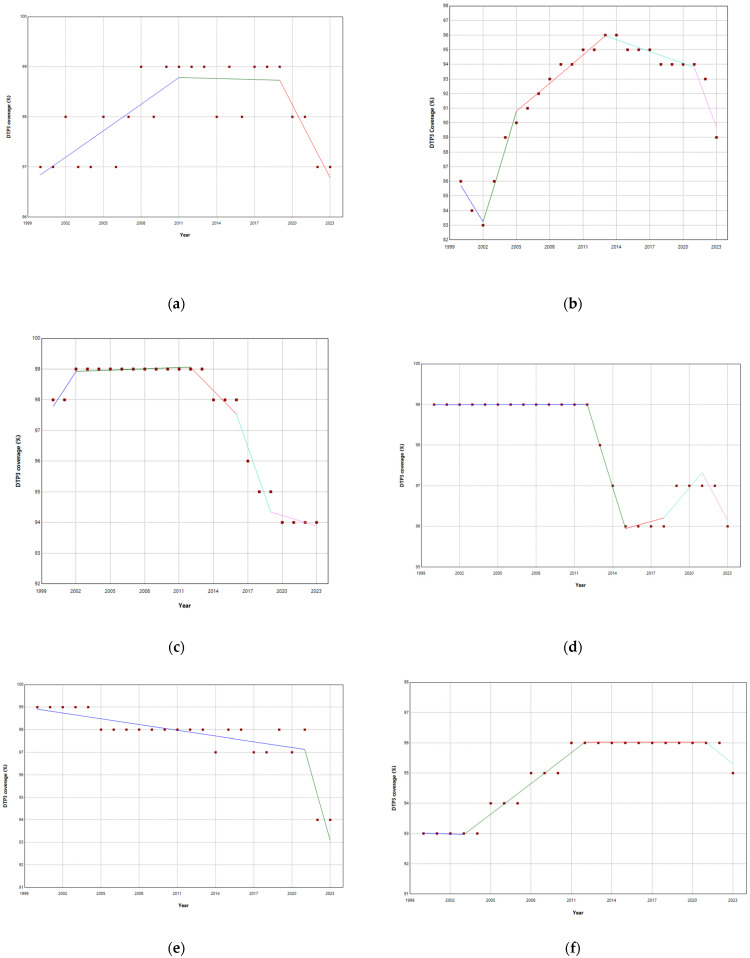
Joinpoint regression of DTP3 in Europe indicates joinpoints at the transitions between colored lines: (**a**) Albania, (**b**) Ireland, (**c**) Poland, (**d**) Slovakia, (**e**) Sweden, and (**f**) Switzerland.

Albania, Belgium, Bosnia, Ireland, Poland, Slovakia, Sweden, and Switzerland had joinpoints close to the pandemic (Table 2, Table S2). The median width of the confidence intervals was 2.8 years, reflecting the variability and uncertainty inherent in the estimates. After the pandemic, Albania, Ireland, Poland, Slovakia, Sweden, and Switzerland presented a negative trend (Figure 5).

**Figure 6:**
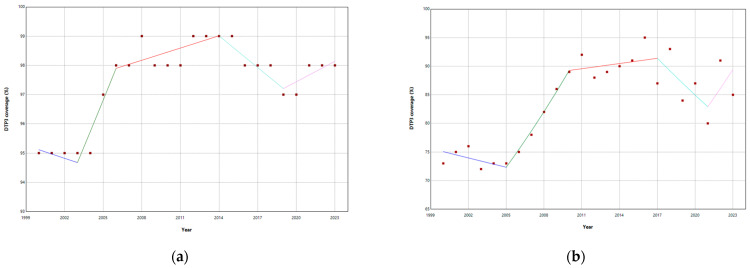
Joinpoint regression of DTP3 in Europe indicates joinpoints at the transitions between colored lines: (**a**) Belgium and (**b**) Bosnia.

In Belgium and Bosnia, where there were joinpoints close to the pandemic, DTP3 grew after the pandemic, with APCs of 0.24% and 3.87%, respectively (Figure 6).

**Figure 7:**
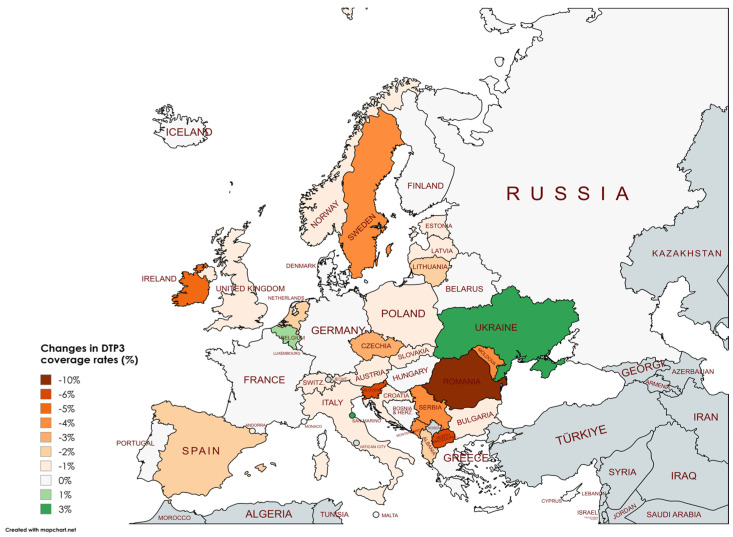
DTP3 coverage rates (%) changes in Europe in 2019 and 2023.

Table 3 shows the variation in DTP3 coverage rates between 2019 and 2023. Most countries experienced slight decreases, often around 1–2%, due to the pandemic. The overall trend indicates a slight, significant decline in vaccination rates across many European countries (Albania, Austria, Belgium, Bulgaria, Croatia, Czechia, Estonia, Ireland, Italy, Latvia, Lithuania, Montenegro, Netherlands, North Macedonia, Norway, Poland, Republic of Moldavia, Romania, Serbia, Slovakia, Slovenia, Spain, Sweden, Switzerland, Ukraine, and United Kingdom). The most significant decrease was observed in Romania, with a drop of 10 percentage points. (Figure 7) Other notable decreases include North Macedonia and Slovenia (−6%), Ireland (−5%), Montenegro, the Republic of Moldova, Serbia, and Sweden with a −4% decrease.

**Figure 8:**
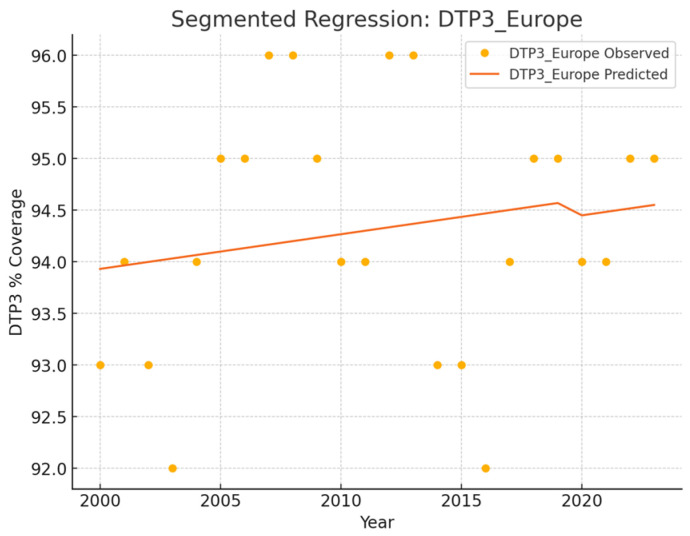
Segmented regression of DTP3 coverage in Europe.

**Figure 9:**
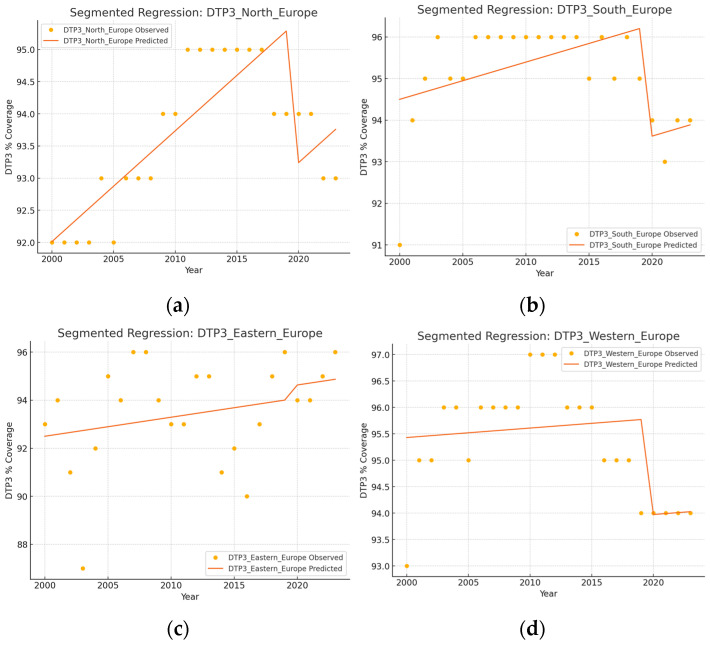
Segmented regression of DTP3 coverage: (**a**) Northern, (**b**) Southern Europe, (**c**) Eastern Europe, and (**d**) Western Europe.

The outcomes of the segmented regression, which examined the relationship between the pandemic and DTP3 coverage, are shown in Table 4. In Europe, the pandemic caused a minimal decrease in vaccination coverage that was not statistically significant (Figure 8). There was a decrease in coverage in Northern (−2.2%), Southern (−2.6%), and Western Europe (−1.8%) (Figure 9).

Table 5 shows the parameters of the segmented regression and includes a column showing the interaction when it exists.

**Figure 10:**
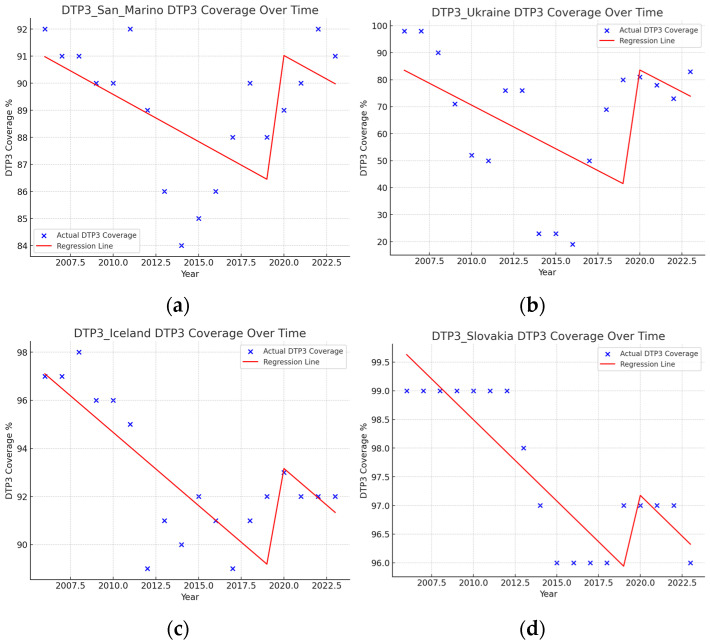
Segmented regression of DTP3 coverage: (**a**) San Marino, (**b**) Ukraine, (**c**) Iceland, and (**d**) Slovakia.

There were five countries where vaccination coverage increased slightly and significantly during the pandemic: Germany, the Russian Federation, San Marino, Iceland, Slovakia, and Ukraine. The biggest increase took place in Ukraine (45.32%) and San Marino (4.92%) (Figure 10).

**Figure 11:**
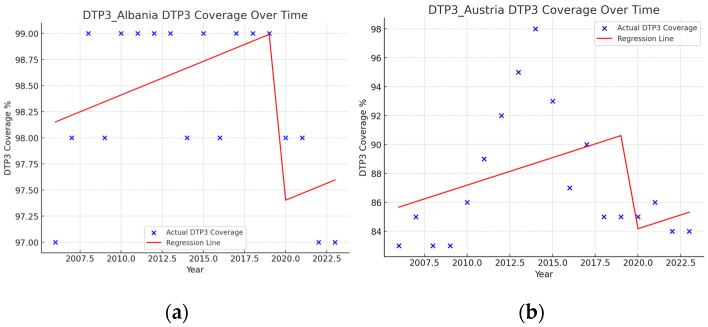

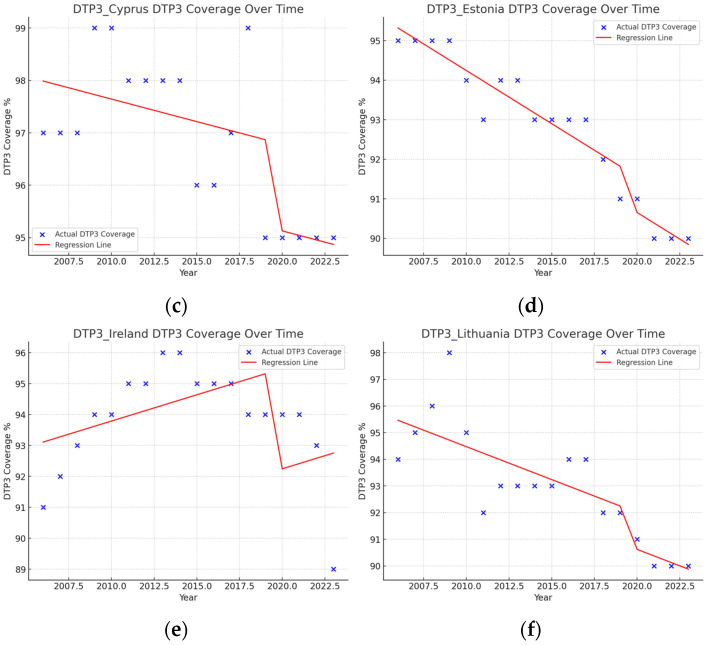
Segmented regression of DTP3 coverage: (**a**) Albania, (**b**) Austria, (**c**) Cyprus, (**d**) Estonia, (**e**) Ireland, and (**f**) Lithuania.

**Figure 12:**
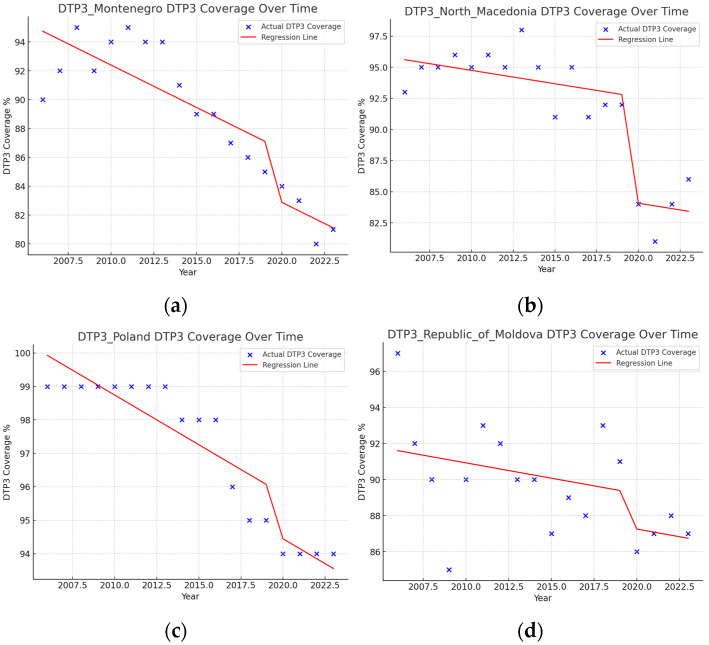

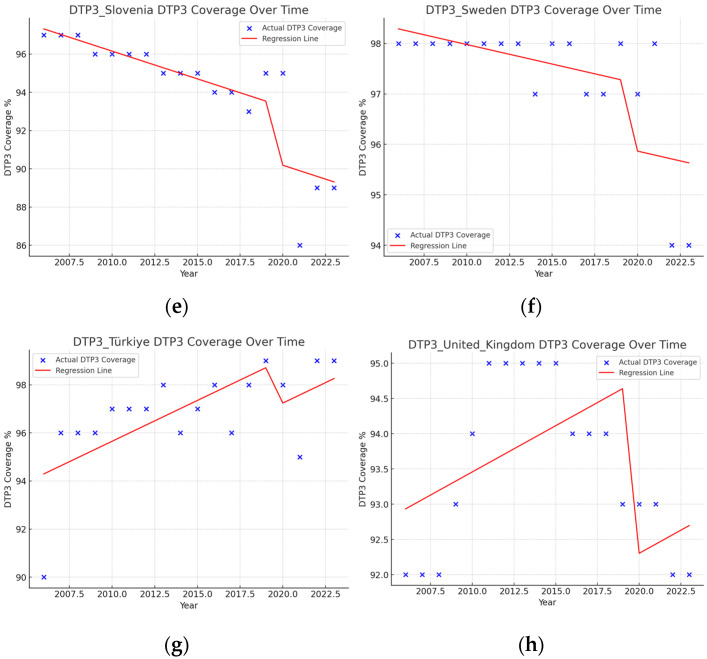
Segmented regression of DTP3 coverage: (**a**) Montenegro, (**b**) North Macedonia, (**c**) Poland, (**d**) Republic of Moldova, (**e**) Slovenia, (**f**) Lithuania, (**g**) Türkiye, and (**h**) United Kingdom.

Segmented regressions show that the pandemic had an immediate negative effect on DTP3 vaccination coverage in Albania (−1.6%), Austria (−6.8%), Cyprus (−1.6%), Estonia (−0.9%), Ireland (−3.2%), Lithuania (−1.3%), Montenegro (−3.6), North Macedonia (−8.5%), Poland (−1.3%), Republic of Moldova (−1.9%), Slovenia (−3%), Spain (−2.4), Sweden (−1.3%), Switzerland (−1%), Türkiye (−1.8%), and the United Kingdom (−2.4%) (Table 5) (Figure 11 and Figure 12). In addition, there were negative interactions between the year and the pandemic in Ireland (−1.8%), Sweden (−1.2%), and Switzerland (−0.4%). This means that, for example, in the case of Switzerland, where the vaccination coverage was practically stagnant with an annual growth of 0.17%, there is a decrease in vaccination coverage of 3.2% in the year of the pandemic and a negative trend begins with a decrease of 1.8% per year. In these three countries, Ireland, Sweden, and Switzerland, the pandemic has changed the trend, with a progressive reduction in DTP3 vaccination coverage.

The results presented in Table 6 demonstrate a strong concordance among the three statistical techniques (Segmented, Chi-Square, and Joinpoint), as the majority of countries show consistent trends, particularly decreases (−) across methods, with limited discrepancies observed.

## 4. Discussion

The COVID-19 pandemic caused disruptions to routine childhood vaccination programs. These disruptions included postponements, altered schedules, and, in some cases, complete suspensions of vaccination services [64,65,66].

### 4.1. Limitations

#### 4.1.1. Data Quality: UNICEF and World Bank

Our study has some limitations that should be acknowledged. One important aspect to address regarding the data used in this study is the methodology by which organizations such as UNICEF and the World Bank collect and verify their information. Understanding these processes and their inherent complexities makes it clear why certain limitations may arise in analyses that depend on publicly available global datasets. While UNICEF, WHO, and the World Bank strive to provide the most accurate and comprehensive data possible through careful harmonization of multiple sources, constraints such as underreporting and data delays can persist, particularly in regions with limited resources.

##### UNICEF

UNICEF relies on various data sources and rigorous verification processes to estimate vaccination coverage, including DTP3 immunization rates. Data are routinely provided by national health authorities, who supply annual reports detailing the number of children vaccinated [67]. These national reports form a foundational layer of UNICEF’s data, ensuring that country-level expertise and locally gathered evidence inform global immunization statistics [68,69].

The WHO and UNICEF produce WHO-UNICEF Estimates of National Immunization Coverage (WUENIC), synthesizing information from national reports, standardized surveys, and other credible data streams [70]. The triangulation aims to produce a more robust and comprehensive picture of vaccination efforts, correcting for potential underreporting or reporting lags. They use deterministic ad hoc estimation rules.

UNICEF conducts and supports multiple surveys and field studies, including large-scale household surveys such as the Multiple Indicator Cluster Surveys (MICS) [71] and partnerships with Demographic and Health Surveys (DHS) [72]. These surveys capture granular data on vaccination uptake at the community and household levels and help reveal coverage disparities that may not be evident through national reports alone. Health facility data collection further strengthens data quality [73]. This multi-layered approach enhances data quality, though some issues with timeliness, accuracy, and completeness may persist, especially in resource-limited settings. By combining multiple data sources and consulting with local experts, UNICEF aims to produce accurate and reliable immunization coverage estimates.

##### The World Bank

The World Bank draws from international databases, country-specific health information systems, and large-scale surveys to produce comparable country-level indicators. These data are vetted and standardized to ensure consistency and comparability across different contexts [74]. It aims to support cross-national analyses that inform policy and funding decisions. However, these processes may still encounter delays in reporting, discrepancies due to limited in-country data collection capacity, and challenges in maintaining data quality during periods of upheaval, such as pandemics.

The World Bank focuses on assessing countries’ readiness for vaccine deployment and monitoring vaccination efforts. During the pandemic, the World Bank deployed the Vaccine Deployment Tracker to identify countries in urgent need of financial and operational support for scaling up vaccine deployment [75]. To collect data on COVID-19 vaccination in Sub-Saharan Africa, the World Bank’s Living Standards Measurement Study (LSMS) program conducted high-frequency phone surveys across multiple countries [76].

#### 4.1.2. Data Quality European Data

The reliance on publicly available data from organizations such as UNICEF and the World Bank might introduce limitations related to data completeness and accuracy, especially in the case of underreporting or data collection challenges during the pandemic due to resource constraints and reporting lags, especially in European low- and middle-income countries, such as some countries in the east or the south of Europe, can further reduce the completeness of data [77,78]. Routine immunization coverage is often measured using administrative data, which relies on registry information of administered doses. However, this method can have biases and inaccuracies, mainly if doses administered outside standard healthcare settings are not recorded consistently [6]. However, as this study focuses on Europe, where countries generally have more robust healthcare infrastructures, stronger data collection systems, and more systematic reporting practices, it is more likely that the quality and completeness of the data used here are relatively high. Consequently, while acknowledging potential limitations in data reporting and completeness is important, the probability of encountering significant data quality issues in this European context is lower compared to regions facing greater resource constraints. However, in Bosnia and Herzegovina, patient file data corroborates the official administrative data, showing consistent vaccination coverage levels, particularly for BCG, DTP, and MMR (measles, mumps, and rubella) vaccines, suggesting that the data for this region may be more reliable despite the challenges mentioned [79].

#### 4.1.3. Intra-Country Disparities, Ethnic Minorities, and Cross-Border Mobility

Another limitation is the use of country regional data, which may mask within-country variations in the impact of the pandemic, particularly in areas with lower healthcare service coverage or among ethnic groups that may reject vaccination or are challenging to reach due to their movement across borders [80,81]. This is especially relevant in regions with diverse healthcare systems such as Eastern Europe. During the 2016–2020 measles outbreak in Romania, certain regions exhibited particularly low vaccination coverage, facilitating the spread of the disease.

**Figure 13:**
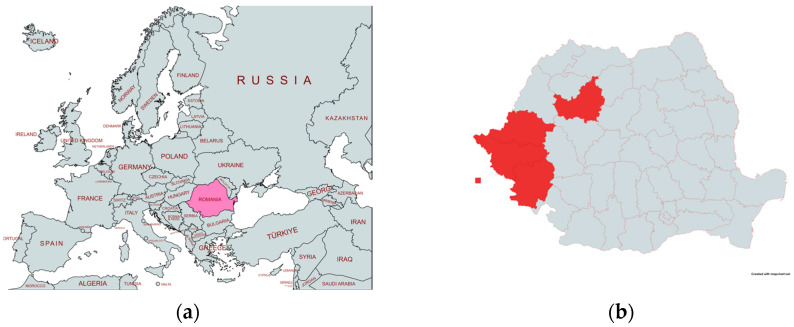
Counties in Romania with low vaccine coverage in red (**a**) Europe and (**b**) Romania.

These regions often included areas with significant minority populations, such as the Roma community, who face multiple barriers to healthcare access. Counties such as Arad, Caras-Severin, Cluj, and Timis in Romania reported suboptimal vaccination rates, with second-dose coverage as low as 29.7% in some areas [82] (Figure 13).

These regions also serve as focal points for disease transmission due to the frequent mobility of the Roma population both within Romania and across borders to countries such as Italy, Spain, France, Germany, and the United Kingdom (Box 1).

Box 1 **Regions with low vaccination coverage in Romania and their impact on vaccine-preventable diseases.**

Counties with Suboptimal Vaccination Rates:

- Arad, Caras,-Severin, Cluj, Timis: These counties exhibited low vaccination rates during recent measles outbreaks, with some areas reporting second-dose coverage as low as 29.7%. These coverage gaps contributed to the spread of diseases within and beyond these regions.

Key Challenges:

- Healthcare Access: Some communities in these regions face structural and logistical barriers to vaccination, such as limited healthcare services, socioeconomic challenges, and mobility.
- Mobility Patterns: The frequent movement of populations within Romania and across borders to countries such as Italy, Spain, France, Germany, and the United Kingdom can complicate vaccination efforts and disease containment strategies.

This transnational movement complicates public health efforts to contain outbreaks and highlights the importance of targeted interventions in regions with low vaccination coverage. Including these areas in analyses can help identify hotspots of vulnerability and inform more effective, localized public health strategies. A summary of these regions and their specific challenges has been included in Box 1 to draw attention to their critical role in the spread of infections and the measles elimination process.

### 4.2. Statistical Methodology: Strengths and Limitations

Although the three statistical methods used identify the same countries, their interpretation differs. Segmented regression indicates if COVID-19 has an immediate effect; the comparison of rates between 2019 and 2023 indicates if the pandemic has an intermediate effect on rates or if the vaccine coverage recovered, while joinpoint indicates if there was a change in the trend of the coverage at a certain point. In our opinion, combining the three methodologies increases the sensitivity and the ability to detect the impact of the pandemic.

The study’s time, which focuses heavily on pre-pandemic trends, might not fully capture the long-term effects of the pandemic on vaccination rates as recovery continues to evolve, so we need to do follow-up studies to know the long-term effects of the pandemic.

Although excluding certain European territories such as the Vatican, Liechtenstein, and other small countries or territories might initially affect the overall results, their small size and unique characteristics do not significantly influence the broader picture of European vaccination coverage.

Lastly, our statistical analysis does not consider the influence of confounding factors, such as concurrent public health interventions or political and economic changes during the pandemic, which may not have been fully accounted for, limiting the interpretation of direct pandemic-related effects.

### 4.3. Country-by-Country Contextual Insights from Available Literature

A study investigating vaccine hesitancy across various regions in the European Union found that higher levels of aggregate vaccine hesitancy were significantly associated with lower immunization rates, particularly for childhood vaccines such as DTP3. This analysis utilized data from the 2019 Eurobarometer survey on vaccine hesitancy and linked it to regional immunization coverage reported by the WHO. The findings suggest that regional differences in vaccination uptake are at least partially attributable to varying public attitudes toward vaccination [83]. The analysis of DTP3 vaccination trends during the COVID-19 pandemic across Europe reveals significant country-specific impacts shaped by unique sociopolitical, economic, and healthcare-related factors. Each country faced distinct challenges and exhibited various trends regarding vaccine uptake, influenced by historical hesitancy levels, the diversion of healthcare resources, public attitudes, and systemic responses to the pandemic. From Albania’s socioeconomic barriers and Austria’s pre-existing hesitancy to Cyprus’s education-linked confidence in vaccination and Serbia’s heightened skepticism fueled by misinformation, the spectrum of experiences underscores the complexity of maintaining routine immunizations during a global health crisis. Despite high immunization rates in some countries, such as Norway, regional or demographic pockets with low coverage present ongoing risks. Understanding these complex trends is essential for tailoring interventions to each context as countries continue working to restore and strengthen immunization programs in the post-pandemic era.

#### 4.3.1. Albania

Albania experienced a significant impact on DTP3 vaccination coverage due to the pandemic; however, even before this, there was already a downward trend in vaccination rates. Despite universal health insurance and increasing healthcare expenditures, childhood immunization coverage declined, even though these services remain free of charge [84,85]. Low coverage was associated with socioeconomic and geographic factors [86], safety concerns, and unreliable information sources. Although vaccine hesitancy is low [87,88], mothers who rely on the internet or social media for health information are less likely to follow vaccination schedules [86]. Although nurses favor vaccination in Albania, more than 20% gave credence to hoaxes such as the association with autism or multiple sclerosis [89].

#### 4.3.2. Austria

In Austria, low vaccine coverage has been limited to DTP3 and measles, making Austria second only to Romania in the number of reported measles cases [90]. The decrease in DTP3 vaccination coverage in Austria during the COVID-19 pandemic may be attributed to several factors related to vaccine hesitancy, which had already been observed before the pandemic. Studies have indicated that vaccine hesitancy and skepticism were significant in Austria, with a considerable proportion of the population expressing concerns about vaccine safety, particularly the fear of side effects [91]. In addition, this hesitancy contributed to a lack of sufficient health literacy and awareness about the importance of routine vaccinations and limited social marketing and promotion efforts to encourage vaccination. Furthermore, many parents in Austria became more hesitant after the pandemic [92]. The pandemic likely exacerbated these issues by straining healthcare systems and diverting attention from routine immunizations to COVID-19-related care.

#### 4.3.3. Belgium

Although the only child compulsory vaccine in Belgium is the vaccine of Poliomyelitis [93,94], Belgium showed an increase in DTP3 coverage and had implemented a pediatric vaccination program, recommended by the Superior Health Council, covering various childhood diseases with immunization coverage rates above 90%, overseen by regional authorities in Flanders, Wallonia, and Brussels [95]. One explanation of this high coverage is that high vaccination coverage rates are positively influenced by urbanization, as seen in countries such as Belgium, Israel, Japan, and Luxembourg, where over 90% of the population resides in urban areas [96,97].

#### 4.3.4. Bosnia and Herzegovina

Gavi, the Vaccine Alliance, is a global organization that helps low- and middle-income countries increase access to life-saving vaccines by providing financial and technical support. Bosnia and Herzegovina transitioned from Gavi support in 2011, but this period saw instability in vaccination coverage [98]. The fluctuations in Bosnia’s vaccination coverage were attributed to several factors, including growing vaccine hesitancy, misinformation spread through social media, and challenges within the health system, such as a shortage of healthcare workers. This instability contributed to an outbreak of pertussis in 2018, during which Bosnia experienced many pertussis cases among unvaccinated children. Thanks to vaccination programs, the number of pertussis cases has been reduced from 93 cases in 2018 to 1 in 2021 [66]. This data is consistent with the increase in DTP3 coverage that we detected in Bosnia.

#### 4.3.5. Bulgaria

In our study, we detected that from 2019 to 2023, Bulgaria experienced a significant decline in DTP3 (diphtheria, tetanus, and pertussis) vaccination coverage, mirroring a broader trend observed in several European countries. This decline could be attributed to multiple factors, including the diversion of healthcare resources during the COVID-19 pandemic, the affluence of refugees [99], and a rise in vaccine hesitancy fueled by misinformation and low vaccine confidence [100]. The pandemic exacerbated existing challenges in Bulgaria’s immunization programs, particularly in underserved communities, where vaccination uptake was already vulnerable. A large-scale study across eighteen European countries before the pandemic found that Bulgaria and Poland had low vaccine confidence [101]. The resulting decrease in DTP3 coverage raises concerns about potential outbreaks of vaccine-preventable diseases and underscores the need for targeted public health interventions to rebuild trust in vaccines and restore routine immunization coverage [102].

#### 4.3.6. Croatia

In Croatia, vaccine-hesitant individuals have reportedly employed tactics to bypass vaccination mandates, including falsifying vaccination records, using alternative substances, and bribing medical personnel [103]. Such behaviors were noted even before the COVID-19 pandemic, especially among parents resisting required childhood vaccinations. A qualitative research Croatian study highlighted similar strategies, such as altering medical documents, visiting a private physician, or offering bribes to pediatricians to evade compulsory vaccination requirements [104]. These challenges underscore the need for multidisciplinary strategies grounded in behavioral science to create more effective vaccination interventions [105]. Studies have observed that higher-education parents have good skills for assessing health information in Croatia, but these skills are lacking in the general population [106].

#### 4.3.7. Cyprus

Our results on Cyprus agree with a study conducted in Cyprus during the COVID-19 pandemic that found that a significant proportion of mothers (57%) reported delaying their children’s vaccinations despite holding a generally positive view of vaccines. Mothers’ knowledge about vaccines varied, with only 13.6% achieving high accuracy in their responses, and knowledge levels were positively associated with higher education and income. A European study performed in 18 countries examined vaccine confidence among parents and found that confidence was notably highest in Portugal and Cyprus, with 78% of Cypriot parents having no hesitation about vaccines [101]. An article highlighted significant COVID-19 vaccine hesitancy among healthcare professionals in Cyprus, especially nurses and midwives [107]. This reluctance toward the COVID-19 vaccine could have contributed to a broader hesitancy toward other vaccines within this group.

Nevertheless, another study showed that in Cyprus, maternal attitudes toward childhood vaccination during the COVID-19 pandemic remained largely positive, with most mothers expressing confidence in vaccine safety, efficacy, and the protection vaccines provide against severe diseases. However, socio-demographic factors influenced these attitudes, with single mothers, those with secondary education, and lower-income households displaying comparatively less favorable views on vaccination [108]. These differences suggest that targeted public health efforts addressing specific socio-demographic barriers may be beneficial. Tailored interventions could enhance vaccine trust and ensure continued support for post-pandemic childhood immunization programs.

#### 4.3.8. Czech Republic

Czechia’s healthcare operates on a mandatory health insurance system; childhood immunization is also compulsory. No vaccination can result in financial penalties and may even affect a child’s ability to attend preschool [109]. Despite high vaccination coverage, the Czech Republic has experienced occasional localized mumps outbreaks due to secondary vaccine failure [110]. Vaccine hesitancy is a notable public sentiment in the Czech Republic [37]. Although we lack specific data on vaccine hesitancy in Czechia, a study on the COVID-19 vaccine revealed that 70.2% of pregnant and lactating women showed a high level of vaccine acceptance [111].

#### 4.3.9. Estonia

In Estonia, adopting a pay-for-performance system for family doctors has significantly improved vaccine coverage rates, as financial incentives encourage higher levels of childhood immunizations, such as the DTP3 vaccine. Factors such as some family doctors not participating in the pay-for-performance quality system reduce vaccine coverage in Estonia [112].

#### 4.3.10. Ireland

Despite high overall self-reported uptake of childhood vaccinations in Ireland, the third dose of diphtheria, tetanus, and pertussis vaccine (DTP3) remains below World Health Organization targets due to vaccine hesitancy. Vaccine hesitancy among parents in Ireland is between 14 and 22% [113]. However, these figures could be even higher because another study found that 26% of respondents in Ireland were hesitant to receive a COVID-19 vaccine, and 9% were resistant [114]. Another study found that slightly over 30% of respondents indicated a possibility of vaccinating their child, while close to 18% stated they would not vaccinate their child [115]. Nevertheless, national surveys indicate parental solid support for vaccinations yet concerns about vaccine safety and side effects are the most common reasons for missed DTP3 vaccinations. Trust in official vaccine information sources predicts vaccine acceptance; increased trust correlates with higher uptake [116,117,118]. Enhancing parental trust in healthcare professionals and public health authorities and addressing safety concerns are crucial strategies for improving DTP3 vaccination rates and achieving target coverage levels in Ireland.

#### 4.3.11. Italy

Italy implemented a mandatory vaccination policy for school admission in 2017 [109]. In Italy, vaccination coverage for mandatory vaccines decreased in 2020 compared to 2019, with declines ranging from 1% to 2.7%. However, coverage for the chickenpox vaccine increased by 2.2% among 7-year-old children during the same period. Recommended vaccinations were moderately affected, with decreases between 1.4% and 8.5%, except for the human papillomavirus vaccine (HPV) in males, the meningococcal conjugate vaccine against serogroups A, C, W, and Y (MenACWY), and rotavirus vaccination, which saw increases of 1.8%, 4.7%, and 9.4%, respectively [119]. A study found that during the pandemic, 44% of parents have had children’s healthcare appointments delayed (29%) or canceled (15%) [120]. In Italy, problems such as those in Croatia, such as vaccine-hesitant individuals bribing medical personnel [121]. The recent pertussis outbreak in Italy (January 2024) underscores the urgent need for enhanced maternal immunization, timely infant vaccinations, and post-exposure prophylaxis to protect vulnerable neonates and reduce severe complications and mortality [122]. There is also the problem of vaccine hesitancy. Research in Italy suggests a strong link between anti-vaccination beliefs and a distrust of science [123]. Additionally, the study indicates that having unrealistic expectations of science can indirectly influence a person’s stance on vaccination.

#### 4.3.12. Latvia

There are scarce studies about vaccine coverage in Latvia [124,125]. The coverage of COVID-19 vaccination in Latvian immigrants in Norway was 42% compared with 92% for immigrants from Vietnam [126].

#### 4.3.13. Lithuania

In Lithuania, one study found that 35.0% of parents did not vaccinate their children. Among the reasons for no vaccination was the cost of paid vaccinations, with 29.3% of parents saying it was too high [127]. Another study found that among vaccine-hesitant parents, information received from healthcare personnel during consultation has a higher impact on their opinion [128].

#### 4.3.14. Moldova

Moldova faces challenges with vaccine hesitancy due to anti-vaccination campaigns and a lack of trust in the healthcare system, particularly regarding vaccine quality. This has led some parents to resist immunization mandates, and reports of falsified documents are being used to circumvent school entry requirements. Additionally, physicians are concerned about their legal protection should children experience adverse events following vaccination, whether real or perceived [129].

#### 4.3.15. Montenegro

A study examining the impact of online media on parental attitudes towards childhood vaccination in Montenegro, Serbia, and Bosnia and Herzegovina revealed that specific parent demographics were more susceptible to online influence. These included women, younger parents (millennials), parents in common-law relationships, and those with larger families [130].

#### 4.3.16. The Netherlands

There has been an increase in parents with negative attitudes toward vaccination in the Netherlands. Some parents are reluctant to vaccinate their children, characterized by unfavorable attitudes toward immunization, skepticism about vaccine effectiveness, safety, and potential side effects; preference for natural infection over-vaccination, and diminished trust in the National Immunization Program [131]. Despite high national vaccination rates, significant clusters of low vaccine coverage persist within specific school communities in the Netherlands. Notably, Orthodox Protestant and Anthroposophic schools exhibit substantially lower vaccination rates, dropping coverage to between 58% and 78% in these institutions [132]. The COVID-19 pandemic notably impacted vaccine-preventable diseases (VPDs) and vaccination rates in the Netherlands. Implementing measures such as social distancing and school closures reduced the incidence of multiple VPDs by approximately 75–97%.

Additionally, the routine administration of vaccines experienced delays for children scheduled between March and September 2020, resulting in an initial decrease in vaccination participation by 6–14% compared to the previous year. Despite these delays, vaccination rates quickly rebounded through catch-up efforts, ultimately maintaining coverage levels with a slight decline of 1–2%. While COVID-19 response strategies effectively lowered the spread of VPDs, the disruption to the infant vaccination schedule was minimal and addressed mainly through subsequent vaccination initiatives [133].

#### 4.3.17. North Macedonia

For years, the Republic of North Macedonia has had a successful immunization system, confirmed by the high population coverage with mandatory vaccines and the evident results regarding eliminating and eradicating infectious diseases. However, there has been stagnation in the population coverage with mandatory vaccines [134]. Negative trends initially emerged due to campaigns led by the anti-vaccine lobby. Later, these issues were compounded by the inconsistent enforcement of mandatory vaccination policies—which effectively became voluntary—and the absence of expected responses from relevant state agencies [134]. In North Macedonia, during the pandemic, there was a decrease in children’s coverage of the influenza vaccine. Data from the 2021/2022 influenza season indicate a substantial decline in vaccination coverage among children aged 6 months to 5 years, decreasing by 87.9% compared to 2020/2021 [135]. Furthermore, fewer than 85% or 80% of specific vaccines in certain territories are worrying [134].

#### 4.3.18. Norway

Even though the coronavirus pandemic has been very demanding regarding resources, according to the Folkehelseinstituttet, Norway had the same high global vaccination coverage as in previous years. This statement disagrees with our findings [136]. Norway’s children’s vaccination program has high and stable vaccine support [136]. In one study made in Australia, the USA, Norway, and the UK, Norway was where the population trusted more in the health authorities. This trust could also influence vaccinations [137]. Another study conducted in the UK, Australia, and Norway found that Norway had the lowest vaccine hesitancy concerning the COVID-19 vaccine [138]. Although vaccine coverage is high in Norway, it is lower among certain groups of immigrants. Children of parents from Somalia, Poland, and Lithuania have lower coverage of MMR, and those from Eastern Europe have lower coverage of DTP and other vaccines than the national average [139,140,141].

#### 4.3.19. Poland

In Poland, the healthcare system is built around compulsory insurance, yet a substantial portion of funding comes from private, primarily out-of-pocket, expenses. While certain vaccinations are required by law, others are merely suggested [142]. Poland, historically known for high childhood vaccination rates due to mandatory immunization laws from the communist era, is experiencing a concerning decline in vaccine uptake. Polish citizens have expressed significant concerns and doubts about the safety and efficacy of vaccines [37]. Increasing vaccine hesitancy has led more parents to refuse or delay mandatory vaccines despite potential fines and legal requirements enforced by family doctors [143]. The growing mistrust in authorities and minorities challenging vaccination laws contributes to this decline, putting public health at risk by potentially falling below essential immunity levels. Another study with Ukrainian economic migrants identified the main barriers experienced in accessing and utilizing Polish vaccination services. Two main barriers to vaccination were identified: challenges related to resources and those unrelated to resources (such as mismatched vaccine schedules, instances of discrimination, the optional status of the HPV vaccine, and a general lack of trust). The lack of sufficient resources, such as the lack of interpreters and healthcare resources, and the unresponsiveness and passivity of the system all contributed to the problem [144]. Regarding the price of vaccines, Ukrainian migrants distinguished between mandatory and “optional”. The optional vaccines were considered less important [144].

#### 4.3.20. Romania

A study in Romania found that 47.8% of postpartum mothers were vaccine hesitant. [145] Another study showed that pertussis vaccine acceptancy (VA) dropped from 85% in 2019 to 44.4% in 2022 [146]. In the case of MMR, Romania has a decreasing trend accelerated by the pandemic [147]. In another study in Romania, vaccine hesitancy was prevalent and negatively affected vaccination rates for infectious diseases, including Invasive Meningitis Diseases (IMD) [37].

#### 4.3.21. Serbia

Under the Law on Population Protection from Communicable Diseases, vaccination against diphtheria, tetanus, and pertussis is required in Serbia [148]. Analysis of immunization trends in Serbia has revealed a significant decline in DTP and DTP3 vaccination coverage between 2000 and 2017 [148]. The evolution of DTP3 vaccination rates in children across the past two decades has likely been influenced by fluctuating levels of vaccine hesitancy among parents, a trend also observed in other European contexts [148,149]. Research indicates that parental attitudes toward vaccines play a significant role in immunization rates, with regions showing higher hesitancy correlating with lower uptake rates for childhood vaccines, such as DTP3. In Serbia, as in other countries, societal attitudes toward vaccination may have been further impacted by the global COVID-19 pandemic, which affected public trust in health interventions. This context underscores the importance of addressing vaccine hesitancy among parents to sustain and improve immunization coverage among children [149]. Serbia experienced measles outbreaks between 2015 and 2019, with a significant number of cases occurring in 2018. In 2018, the number of measles cases in Serbia tripled, making it the second highest in Europe after Ukraine [149]. Most cases involved unvaccinated children younger than five due to decreased MMR vaccination coverage below the recommended threshold [150]. During the pandemic, there was a decline in MMR coverage rates in Serbia, underscoring the pandemic’s broader impact on routine childhood vaccinations, paralleling trends seen with other essential vaccines such as DTP3 [151]. These disruptions emphasize the critical need for catch-up strategies to restore coverage levels and safeguard against preventable disease outbreaks.

During the COVID-19 pandemic, skepticism about vaccines in general and the COVID-19 vaccine became widespread in Serbia [35]. Public opposition to vaccination was observed in Serbia [37].

A growing public health challenge is posed by people becoming increasingly skeptical of the efficacy and safety of vaccines. A study in Serbia found that vaccine hesitancy and refusal are growing concerns [149]. This hesitancy is linked to distrust of medical science and institutions and a belief in conspiracy theories. The authors suggest that the abundance of misinformation on social media and the internet and distrust in official institutions contribute to this problem. Parents who endorse anti-vaccine conspiracy theories show a lower intention to vaccinate their children. The impact of vaccine refusal and the spread of misinformation can have harmful, even deadly, consequences [35].

A study in Serbia found that pediatricians play a key role in parents’ decisions to vaccinate their children with the MMR vaccine [152]. Other factors associated with higher MMR vaccination rates included having two children, previous vaccination of the child, and a higher parental vaccination knowledge score. Also, research on HPV vaccination in Serbia demonstrated that a recommendation from a pediatrician was the strongest motivator for parents to vaccinate their children [153]. Most parents rely on pediatricians as their primary source of immunization information, highlighting the importance of pediatricians’ knowledge and attitudes toward vaccines in shaping parental decisions.

#### 4.3.22. Slovenia

In Slovenia, vaccine hesitancy has been a factor in lower immunization rates against IMD [37]. A study involving nursing students in Slovenia, Poland, and Serbia highlighted the influence of healthcare professionals’ attitudes on public vaccination decisions. Factors such as perceived vaccine benefits, trust in institutions, and vaccine effectiveness significantly impacted students’ intentions to vaccinate and advise others to do so. The study found that greater trust in institutions and healthcare providers was associated with increased vaccination willingness [154].

#### 4.3.23. Spain

The COVID-19 pandemic significantly disrupted pediatric vaccination coverage in Spain, impacting the continuity of immunization schedules, particularly during the early stages of lockdown when healthcare services were suspended. Confinement measures, closure of health facilities, and public fear of SARS-CoV-2 transmission contributed to reduced access to vaccination, mainly affecting children under two years of age. The COVID-19 pandemic has significantly impacted child vaccination coverage in Spain, affecting routine immunizations and campaigns. The shift to telemedicine, fear of infection, and restrictions on physical attendance at health centers reduced vaccination rates, with declines between 5% and 60% across regions, varying by age and vaccine type [155]. While essential vaccines such as DTaP (diphtheria, tetanus, and acellular pertussis) in children under 15 months were somewhat maintained, declines included an 8–13% drop for primary doses at 2 and 4 months, 15% for 11 month boosters, 12% for MMR and meningococcal ACWY at 12 months, and 20% for varicella at 15 months. Unfunded vaccines saw the steepest drops, with meningococcal B down by 68.4% in April in Valencia and 39% in total doses in Andalusia. Vaccination for pregnant women remained largely unaffected [155]. School-based immunizations were suspended, and only certain critical vaccines, such as DTP3 for pregnant women, were prioritized [155]. Although vaccination rates for infants recovered after initial disruptions, coverage for booster vaccines lagged, with MMR coverage notably falling below the threshold required to prevent measles outbreaks. In response, Spanish health authorities issued urgent recommendations to prioritize essential vaccinations, but regional disparities persisted in vaccination rates [156]. In Catalonia (Spain), vaccination coverage for DTP3 and other routine childhood vaccines decreased despite efforts to maintain essential immunization services. During the lockdown, healthcare services focused on vaccinating children under 15 months and vulnerable populations, but logistical challenges and restrictions hindered access for many. While some vaccines, such as the PCV13, recovered, others, including DTP3, remained below pre-pandemic levels, highlighting the pandemic’s disruptive effect on vaccine coverage.

Our study detected decreased DTP3 coverage in Spain, but some regional studies describe increases. Contrary to what might be thought in Central Catalonia, Spain, the COVID-19 pandemic increased overall influenza vaccination, reducing the usual discrepancy between native and immigrant child vaccination rates [157]. In the same way, a study performed in Cantabria (Spain) showed that, contrary to expectations, the COVID-19 pandemic did not significantly affect routine childhood vaccination rates, as these remained stable between the pre-pandemic and pandemic cohorts [158]. However, a substantial increase was observed in non-routine vaccinations, with mothers being three times more likely to opt for these additional vaccines during the pandemic period [158].

Another problem is vaccine hesitancy. A study in Spain on COVID-19 vaccine hesitancy found that it was influenced by concerns over vaccine safety, distrust in its expedited development, and perceived experimental nature. The hesitant commonly believed that vaccines were financially motivated or ineffective. Demographic factors such as younger age groups (mainly 18–40 years) showed lower uptake, partly due to structural issues and distrust in healthcare information sources, particularly among those relying on social media for information [159]. Another factor was believing in or using alternative therapies [160]. High trust in Spain’s healthcare system, a culture supportive of vaccination, and universal healthcare access likely contributed to the overall success of vaccination campaigns [159]. Maternal education, employment, and income influenced vaccination choices, particularly for non-routine vaccines, which were more frequently chosen by higher-income and educated mothers [158].

#### 4.3.24. Sweden

The COVID-19 pandemic influenced child immunization rates in Sweden, with various factors impacting vaccine uptake. Firstly, lockdowns and reallocating healthcare resources disrupted routine immunization services, leading to delays in childhood vaccinations. There was also increased vaccine hesitancy, partly fueled by misinformation and fears related to COVID-19 vaccines, which may have impacted overall trust in immunization programs. Similar to other European countries, DTP3 coverage faced setbacks in Sweden, impacting pertussis immunity. Additionally, post-pandemic immunity debt, resulting from reduced pathogen exposure, has been associated with increased susceptibility to diseases such as pertussis, underscoring the importance of maintaining high immunization coverage to protect against resurgence in vaccine-preventable diseases.

#### 4.3.25. Switzerland

A study on Swiss childhood vaccination from 2012 to 2021 highlights critical factors affecting vaccination rates, particularly the impact of the COVID-19 pandemic and regional variations. Despite high initial coverage, booster doses are often missed or delayed, resulting in an overall coverage below the Swiss Federal Office of Public Health target of 95%. The significant regional differences, with lower coverage in German-speaking and rural areas, are possibly due to varying vaccine hesitancy and logistical barriers [161]. Another study found that most Swiss parents intend to vaccinate their children more frequently, especially in Latin Switzerland, which was more affected by the pandemic than the German-speaking regions [162]. Amendments to the vaccination schedule in 2019, switching to a 2+1 scheme for the DTP-IPV-Hib vaccine (diphtheria, tetanus, pertussis, inactivated polio, and Hemophilus influenza type b), led to improvements in timeliness and coverage. However, the pandemic’s effects were minimal in disrupting vaccination progress, with some vaccines, such as MMR, experiencing delays due to possible vaccine hesitancy or reluctance to administer multiple vaccines concurrently [161]. Another study conducted in Geneva, Switzerland, on COVID-19 found that parental willingness to vaccinate children was related to socioeconomic factors that play a critical role: parents with secondary or primary education levels and those with middle or low household incomes show greater reluctance to vaccinate their children [163].

#### 4.3.26. Türkiye

The COVID-19 pandemic led to substantial disruptions in routine childhood immunizations globally, and Türkiye was no exception. Initial lockdowns and movement restrictions made access to healthcare facilities challenging, leading to delays in vaccinations and coverage rates, including for the DTP3 vaccine [164]. Factors such as parental fear of exposing children to COVID-19 during healthcare visits, logistical challenges in vaccine distribution, and reduced healthcare accessibility have contributed to declines in immunization coverage [165], primarily due to restricted healthcare access and parental hesitancy influenced by concerns over virus transmission during hospital visits. In Ankara, vaccination rates for children under 24 months decreased by approximately 2–5%, with the most pronounced decline observed in vaccines scheduled for children over 18 months [166]. This decline aligns with broader global patterns observed in various countries, where vaccination coverage fell by over 10% in the first months of the pandemic [164]. The COVID-19 pandemic significantly impacted public perspectives on vaccination in the Republic of Türkiye, with a notable increase in vaccine hesitancy. In one study, 19.6% of participants reported hesitancy regarding routine childhood vaccines, a figure aligned with global studies that indicate similar hesitancy rates across different populations [167]. A study conducted at Trakya University Hospital in Edirne, Türkiye, showed that parental hesitancy towards routine childhood vaccines significantly increased as the pandemic progressed [168]. Among parents surveyed during two COVID-19 peak periods, vaccine hesitancy rose markedly, from 10.6% after the first peak to 20% after the second. Additionally, mean scores on the WHO Vaccine Hesitancy Scale were higher among parents after the second peak, indicating increased hesitancy. This shift is the overall rise in childhood vaccine hesitancy during the pandemic, suggesting a need for proactive public health communication strategies to maintain high vaccination coverage in Türkiye [169]. Another survey of parents of hospitalized children in Türkiye found vaccine hesitancy in 9.38% of the parents [164].

#### 4.3.27. Ukraine

In Ukraine, there have been recent public health sector reforms that may affect vaccination uptake [170]. A study previous to the 2022 war found that prevailing mistrust in the healthcare system and limited access to trustworthy information contributed to the vaccine-related concerns expressed by many parents. [171] In addition, Roma parents encountered systemic obstacles to obtaining vaccines. Vaccine hesitancy in Ukraine is also a problem among refugees [172]. It also posed challenges to achieving high vaccination coverage for IMD [37].

The conflict in Ukraine has led to a significant displacement of children and adolescents, resulting in serious public health challenges in host countries. Many Ukrainian children arrive with low vaccination rates, including for preventable diseases such as measles, polio, and COVID-19, raising concerns about potential outbreaks. Vaccine hesitancy remains prevalent among Ukrainian refugees, further compounding efforts to catch up on vaccinations [173,174,175]. Addressing these issues requires robust vaccination campaigns, public health education, and coordination among healthcare providers to ensure timely vaccinations, as well as psychological and medical support to mitigate the trauma and complex healthcare needs of these young refugees [172].

#### 4.3.28. United Kingdom

The United Kingdom (UK) has a nationalized healthcare system in which vaccines are recommended and provided to the public through doctors’ offices [176]. The country has a significant history of debates surrounding childhood vaccinations, especially concerning the MMR vaccine [177,178,179,180,181,182]. The influence of previous controversies, such as the Wakefield study linking MMR vaccines to autism, still resonates, with parents citing these fears [183]. The vaccine uptake dropped by approximately 20% in the initial weeks of the lockdown.

The COVID-19 pandemic disrupted routine healthcare services in the UK, including child immunizations, as resources were redirected to COVID-19 responses. Lockdowns, fear of infection, and changes in healthcare accessibility led to delays and reductions in routine vaccination [183]. A study found that 47% of parents have had a children’s health care appointment delayed (24%) or canceled (23%) [120]. Public messaging focused on encouraging the public to “stay at home”, which inadvertently led parents to avoid healthcare facilities, fearing exposure to COVID-19 and a desire not to overburden the National Health Service (NHS). Reports indicated that 60% of families considered postponing or canceling immunizations due to these concerns [4].

During the pandemic, the acceptance of vaccinations decreased in the UK. A study found that 25% of respondents in the UK were hesitant to receive a COVID-19 vaccine, and 6%, respectively, were resistant. This hesitancy aligns with general trends of vaccination resistance observed for other vaccines, indicating an underlying public health concern that extends beyond COVID-19 and potentially impacts routine childhood vaccinations. In the UK, vaccine hesitancy and resistance are influenced by sociodemographic factors, including age, income level, and gender. Women, younger adults, and those in lower income brackets were found to be more vaccine-hesitant [114]. The COVID-19 pandemic highlighted a global challenge of misinformation that directly affects public willingness to vaccinate. Findings suggest that misinformation reduces vaccination intent by approximately six percentage points in the UK [7]. Misinformation affects different sociodemographic groups in varying ways, with females, lower-income groups, and some ethnic minorities more susceptible to altered vaccination intentions [7]. Parents from these demographics might similarly display hesitancy toward child immunizations if they perceive vaccination as unsafe or unnecessary [184].

In the UK, political affiliation influenced vaccine intentions, with Labour Party supporters showing higher vaccination intentions than Conservative Party supporters. This difference is partially attributed to the UK’s regulatory media environment, which is less polarized and is overseen by Ofcom, supporting more balanced public health messaging. This context suggests that political and media landscapes impact vaccine perceptions differently across regions [8]. In the UK, reliance on legacy media (such as broadcast TV and newspapers) correlated positively with vaccine intentions, while social media, particularly platforms such as Facebook and YouTube, correlated negatively. This negative association on social media may stem from misinformation and conspiracy theories regarding vaccines, especially COVID-19, which saw high engagement on these platforms [185]. Misinformation videos on COVID-19 vaccines spread primarily via Facebook and YouTube shares, and this content often received higher engagement than factual information.

Thus, public health campaigns in the UK may benefit from focusing on regulated media while addressing misinformation on social platforms [8]. Socioeconomic and ethnic factors also influenced vaccine intentions in the UK. Lower vaccine intentions were associated with minority ethnic groups and lower-income households.

Disparities in healthcare access exacerbated the reductions in vaccination coverage. Vaccination rates are typically lower in socioeconomically disadvantaged areas and among minority ethnic groups, who were disproportionately affected by COVID-19 and may hesitate to seek healthcare due to higher perceived risks. Areas with higher deprivation levels and households with lower incomes experienced more significant disruptions in immunization schedules due to increased logistical barriers and healthcare access challenges during COVID-19. Families in these communities were often hesitant or unable to attend catch-up campaigns when clinics reopened [183]. This situation also intersects with challenges in public communication, as health information is often not tailored to reach families with limited English proficiency, literacy, or digital access, such as recent migrants [4].

### 4.4. Strategies for Enhancing DTP3 Vaccination Coverage in Europe Amidst and Beyond COVID-19 Challenges

The COVID-19 pandemic significantly impacted routine childhood vaccination programs across Europe, including those for DTP3. In response to vaccine hesitancy and fluctuating coverage rates, several European countries have implemented mandatory or incentive-based vaccination policies, which have generally proven effective in increasing vaccine uptake and reducing the incidence of vaccine-preventable diseases. Studies from countries such as France and Italy, which adopted stricter immunization policies before the pandemic, show that these mandates can sustain or even improve vaccination coverage despite the challenges posed by COVID-19 [119].

Immunization programs are very beneficial for society and the health care system. The Polish immunization program is very effective. Each euro invested produces a return of two and seven euros from the perspective of health care and society [186]. Ensuring equitable access to childhood vaccinations, such as the DTP3 vaccine, is essential, as systemic biases against prioritizing children’s health can hinder fair healthcare access and compromise their well-being, especially when vaccine hesitancy and safety concerns are prevalent among parents [187]. A holistic approach is essential to improve DTP3 vaccination coverage across Europe, integrating immunization into broader child health services and addressing practical barriers such as cost, accessibility, and scheduling, alongside fostering trust through enhanced e-health solutions that connect families with tailored support and information systems [188]. Expanding vaccination efforts to non-primary care settings for children could significantly improve immunization rates, especially among underserved populations. Similar to adults, parents often appreciate the convenience, accessibility, and reduced costs of receiving vaccines outside traditional clinics, such as community centers, schools, or mobile clinics. These locations can reduce logistical barriers, particularly for parents facing time or transportation challenges. However, parental concerns about vaccine safety, fear of side effects, and a strong preference for vaccinations administered by a primary pediatrician remain common barriers. Addressing these concerns through trusted community-based nurses, clear information about vaccine benefits, and flexible vaccination opportunities could support parents’ decisions to vaccinate their children. Broadening immunization access through non-traditional settings thus has the potential to enhance child vaccination rates, reduce disparities, and support public health goals for childhood immunization [189].

Home vaccination by nurses could be a feasible strategy to combat vaccine hesitancy and improve coverage. Home visits for vaccination have effectively boosted coverage among hard-to-reach populations, targeting under-vaccinated children in socioeconomically deprived areas. Offering home vaccinations via a trained team can increase coverage quickly [190]. To counteract this decline, healthcare professionals, especially nurses, should be encouraged to actively reach out to parents and pregnant women, especially in communities with historically low vaccination uptake.

Efforts include clear messaging to reassure families that vaccination services are safe and essential. Some healthcare facilities adapted to the pandemic by establishing drive-in vaccine clinics and other measures that minimize in-person contact [4]. Misinformation exposure often occurs in online environments where individuals can encounter exaggerated vaccine risks. Thus, understanding these digital ecosystems and improving the visibility of reliable information is crucial for protecting child vaccination rates. Misinformation spreads more rapidly than factual information, a factor public health agencies must consider when designing countermeasures. Legacy media use, including broadcast and print media, has been linked with higher vaccine acceptance than social media, which has often hosted vaccine-skeptical narratives. During the pandemic, high reliance on social media for health information was correlated with greater hesitancy, whereas engagement with traditional media was associated with a more positive outlook on vaccines. This dichotomy suggests that public health campaigns must balance digital engagement with efforts to direct audiences toward reliable sources, especially for immunization information aimed at parents [8].

These points reflect how the challenges observed with COVID-19 vaccine misinformation may have broader implications for routine child immunization programs, emphasizing the need for vigilant public health strategies to sustain immunization rates among children.

The pandemic disrupted healthcare services globally, leading to delays in routine child immunizations. With vaccine hesitancy possibly fueled by pandemic-related distrust, the risk of a continued decline in child immunization rates is high. There is a need for renewed efforts to prioritize routine vaccinations post-pandemic, ensuring vaccine hesitancy does not impede the return to pre-pandemic immunization coverage levels.

Government support and investment in primary care infrastructure, community health initiatives, and more robust public health surveillance are essential to monitor and address vaccination gaps. Ensuring robust call-and-recall systems for missed vaccines, improving safety in clinical settings, and strengthening public trust in primary care is pivotal to restoring immunization levels and protecting child health in the long term [4].

Our paper has revealed critical vulnerabilities in routine immunization programs. Ensuring resilience in these programs during future health emergencies will require addressing the socioeconomic factors that drive disparities in vaccine access. Addressing these challenges should focus on enhancing accessibility through mobile vaccination units, tailored health communication strategies, and local engagement with community leaders. Cross-border collaboration between Romania and other European countries is critical to managing the transnational spread of vaccine-preventable diseases.

### 4.5. Strengthening DTP3 Vaccination Coverage in Europe Amidst and Beyond COVID-19

Achieving and maintaining high DTP3 vaccination coverage requires a multipronged approach that addresses both demand- and supply-side challenges, particularly during COVID-19 disruptions. Targeted public health campaigns are needed to confront vaccine hesitancy and misinformation by providing accurate, culturally sensitive information on the safety and benefits of childhood vaccinations. These campaigns can enhance their credibility and outreach by engaging trusted community leaders and healthcare providers and leveraging digital platforms and social media to counteract the spread of false information.

Improving service accessibility is paramount. Expanding clinic operating hours, integrating vaccinations into routine pediatric care, and deploying mobile vaccination units can reduce logistical barriers, especially in underserved communities. Universal adoption of digital vaccination records and strengthened real-time monitoring systems can guide timely, data-driven interventions when coverage gaps emerge.

Policy measures should also incentivize timely vaccination through financial or non-financial rewards—such as tax benefits, childcare support, or community recognition—ensuring that proactive health behaviors are recognized and encouraged. Equally important is equipping healthcare professionals with up-to-date training in both clinical protocols and effective communication strategies, allowing them to address parental concerns more confidently. Finally, investing in robust healthcare infrastructure, including vaccine stockpiles, resilient supply chains, and a trained reserve workforce, will help sustain vaccination efforts during public health emergencies, ensuring that Europe’s vaccination programs remain resilient and effective beyond the pandemic.

### 4.6. Areas for Future Research

Future research should focus on understanding the dynamics of vaccine hesitancy by exploring sociocultural and psychological factors that influence public attitudes toward vaccinations across different regions. Investigating the role of misinformation and developing tailored interventions to address specific community concerns will provide actionable insights. Additionally, longitudinal studies are needed to evaluate the long-term impacts of the COVID-19 pandemic on immunization programs. These studies can determine whether declines in vaccination rates were temporary or indicative of lasting trends and assess the effectiveness of post-pandemic recovery interventions.

The role of digital technology in immunization efforts warrants further exploration. Research should assess how tools such as electronic health records and mobile health applications can improve vaccination tracking, communication, and coverage. Comparing the effectiveness of various vaccination policies, such as mandatory laws, financial incentives, and public awareness campaigns, across diverse healthcare systems and vaccination challenges can help identify best practices. Furthermore, it is essential to study disparities in vaccination coverage among marginalized groups, including ethnic minorities and immigrant populations, to design targeted solutions that reduce these inequities.

Another critical area for investigation is the concept of “immunity debt,” resulting from reduced pathogen exposure during the pandemic. Understanding its implications for vaccination policies and future disease outbreaks can guide public health strategies. Finally, research should explore the relationship between healthcare system resilience and vaccination coverage during crises, identifying key features that help maintain vaccination rates and prevent disruptions. These research efforts will provide the evidence base needed to strengthen immunization programs and address the challenges highlighted by the COVID-19 pandemic.

## 5. Conclusions

The COVID-19 pandemic has profoundly disrupted DTP3 vaccination programs across Europe, exposing significant regional disparities in coverage and recovery. While overall vaccination rates in Europe remain high, several countries have experienced declines, with regions such as Eastern Europe facing more significant challenges in regaining pre-pandemic coverage levels compared to more resilient regions such as Southern and Western Europe. These variations underscore the need for targeted public health strategies to address vaccine hesitancy, logistical barriers, and systemic inequities exacerbated by the pandemic.

The pandemic has introduced immediate and sustained challenges to routine immunization efforts, with statistical models revealing shifts in vaccination trends in countries such as Ireland, Sweden, and Switzerland, which experienced reductions in coverage. Conversely, countries such as Ukraine and San Marino reported notable improvements, highlighting the importance of localized healthcare strategies and robust public health interventions.

To mitigate the “immunity debt” caused by pandemic-related disruptions and to protect vulnerable populations from vaccine-preventable diseases, measures should be taken to enhance trust in vaccination, leverage community healthcare resources, and combat misinformation. While some healthcare systems have demonstrated resilience, sustained efforts to reinforce healthcare infrastructure and adapt immunization programs are essential to ensure equitable access and uptake, safeguard against future public health crises, and protect global health achievements.

## Figures and Tables

**Table 1:** Joinpoint analysis for the DTP3 vaccination regional rates in Europe, 2000–2023.

Region	Number of Joinpoints (Joinpoints)	APC Total Period	APC1	APC2	APC3	APC4	APC5
Europe	0	0.03	-	-	-	-	-
North	3 (2008, 2011, 2016)	0.11 *	0.18 *	0.65 *	0.02	−0.31 *	−
South	4 (2002, 2009, 2018, **2021**)	−0.02	2.05 *	0.12 *	−0.06	−0.81 *	0.50
East	0	0.11	−	−	−	−	−
West	4 (2002, 2008, 2011, **2020**)	−0.12	1.17 *	0.06	0.41	−0.37 *	0.03

Bold indicates Joinpoint close to pandemic; * Annual Percent Change (APC); *p* < 0.05.

**Table 2:** Joinpoint analysis for the DTP3 vaccination rates in Europe, 2000–2023.

Country	Number of Joinpoints (Joinpoints)	APC Total Period	APC1	APC2	APC3	APC4	APC5
Albania	2 (2011, **2019**)	0.04	0.18 *	−0.01	−0.50 *		
Andorra	0	0.06					
Austria	3 (2009, 2014, 2017)	0.19	0.1	3.0 *	−3.8 *	−0.5	
Belarus	2 (2003, 2006)	0.50	−13.3 *	11.6 *	−0.2		
Belgium	4 (2003, 2006, 2014, **2019**)	0.12	−0.15	1.12 *	0.14 *	−0.37 *	0.24 *
Bosnia	4 (2005, 2010, 2017, **2021**)	0.96	−0.74	4.30 *	0.34	−2.41 *	3.87 *
Bulgaria	1	−0.21 *	0.76	−0.31 *			
Croatia	1 (2008)	−0.12	0.41 *	−0.34 *			
Cyprus	1 (2011)	−0.10	−0.14	0.31 *			
Czechia	1 (2011)	−0.15	0.14	−0.41 *			
Denmark	3 (2004, 2007, 2018)	0.13	−0.38	−2.88 *	0.91 *	−0.06	
Estonia	1 (2007)	−0.21 *	0.33 *	−0.37 *			
Finland	2 (2014, 2017)	−0.46 *	0.06	−3.11 *	0.17		
France	4 (2003, 2006, 2013, 2016)	−0.09 *	0.04	0.58	0.02	−1.00	−0.00
Germany	3 (2003, 2010, 2017)	−0.21 *	2.18 *	−0.05	−0.76 *	0.02	
Greece	2 (2003, 2007)	0.33	1.71 *	1.36	−0.00		
Hungary	0	0.00	0.27	−2.55 *	0.18		
Iceland	2 (2009, 2012)	−0.30 *	0.27	−2.55 *	0.18		
Ireland	4 (2002, 2005, 2013, **2021**)	0.40 *	−1.49 *	2.95 *	0.69 *	−0.28 *	
Italy	1 (2003)	0.09	2.85 *	−0.08			
Latvia	1 (2011)	−0.01	−0.55 *	0.46 *			
Lithuania	1 (2009)	−0.21 *	0.14	−0.40 *			
Luxembourg	0	0.01					
Malta	3 (2005, 2008, 2012)	0.60	−0.77	−9.17 *	9.46 *	−0.06	
Monaco	0	0.00					
Montenegro	1 (2011)	−0.91 *	0.94 *	−1.43 *			
Netherlands	0	0.29					
North Macedonia	1 (2013)	−0.48 *	0.21	−1.47 *			
Norway	0	0.29					
Poland	2 (2013, **2020**)	−0.23 *	0.06	−0.76 *	−0.03		
Portugal	0	0.14					
Republic of Moldova	0	−0.50					
Romania	0	−0.83					
Russian Federation	2 (2007, 2011)	0.01	0.36 *	−0.32 *	0.00		
San Marino	1 (2015)	−0.40 *	−0.92 *	0.90 *			
Serbia	0	−0.02					
Slovakia	3 (2012, 2015, **2021**)	−0.15	0.00	−1.10 *	0.23 *	−0.50 *	
Slovenia	1 (2008)	−0.16	0.86 *	−0.57 *			
Spain	1 (2012, 2016)	−0.16 *	1.24	−0.07	−0.61		
Sweden	1 (**2021**)	−0.14 *	−0.09 *	−1.94 *			
Switzerland	3 (2003, 2012, **2021**)	0.16 *	−0.01	0.36 *	0.00	−0.45 *	
Türkiye	2 (2003, 2006)	0.89 *	−6.17 *	9.35 *	0.18		
Ukraine	3 (2012, 2015, 2018)	−2.63	−3.75	−35.32 *	56.47 *		
United Kingdom	2 (2008, 2012)	0.11 *	0.18	0.91 *	−0.34 *		

Bold indicates Joinpoint close to pandemic; * Annual Percent Change (APC); *p* < 0.05.

**Table 3:** DTP3 coverage rates (%) changes in European countries between 2019 and 2023.

	2019		2023			
Country	DTP3 (%)	Births (n)	DTP3 (%)	Births (n)	Change in DTP3 (%)	*p* ^†^
Albania	99	29,773	97	28,538	−2	<0.001
Andorra	99	590	98	569	−1	ns
Austria	85	84,744	84	84,132	−1	<0.001
Belarus	98	93,218	98	86,154	0	ns
Belgium	97	116,136	98	116,823	1	<0.001
Bosnia and Herzegovina	73	29,754	73	26,279	0	ns
Bulgaria	93	61,637	92	55,495	−1	<0.001
Croatia	94	36,700	93	33,537	−1	<0.001
Cyprus	95	13,237	95	12,348	0	ns
Czechia	97	109,738	94	99,736	−3	<0.001
Denmark	97	60,455	97	65,003	0	ns
Estonia	91	14,158	90	13,102	−1	0.005
Finland	91	45,790	91	47,143	0	ns
France	96	701,300	96	671,363	0	ns
Germany	91	771,867	91	755,129	0	ns
Greece	99	81,148	99	76,080	0	ns
Hungary	99	92,375	99	104,762	0	ns
Iceland	92	4402	92	4553	0	ns
Ireland	94	60,463	89	56,728	−5	<0.001
Italy	96	418,113	95	406,243	−1	<0.001
Latvia	99	18,693	98	15,996	−1	<0.001
Lithuania	92	28,075	90	25,605	−1	<0.001
Luxembourg	99	6167	99	6724	−2	ns
Malta	98	4318	98	4863	0	ns
Monaco	99	331	99	323	0	ns
Montenegro	85	7429	81	6773	0	<0.001
Netherlands (Kingdom of the)	94	169,827	92	182,435	−2	<0.001
North Macedonia	92	20,447	86	19,769	−6	<0.001
Norway	97	54,384	96	54,464	−1	<0.001
Poland	95	382,527	94	423,741	−1	<0.001
Portugal	99	85,439	99	79,465	0	ns
Republic of Moldova	91	40,266	87	49,667	−4	<0.001
Romania	88	203,903	78	214,232	−10	<0.001
Russian Federation	97	1,486,730	97	1,375,609	0	ns
San Marino	88	209	91	199	3	ns
Serbia	97	69,672	93	65,195	−4	<0.001
Slovakia	97	57,020	96	62,451	−1	<0.001
Slovenia	95	19,865	89	18,520	−6	<0.001
Spain	95	354,681	93	351,908	−2	<0.001
Sweden	98	114,109	94	113,685	−4	<0.001
Switzerland	96	85,930	95	86,100	−1	<0.001
Türkiye	99	1,303,201	99	1,214,053	0	ns
Ukraine	80	353,538	83	184,455	3	<0.001
United Kingdom	93	709,079	92	677,708	−1	<0.001

ns = no significant, ^†^ chi-square test.

**Table 4:** Segmented regression of DTP 3 coverage in European Regions.

Region	Intercept	Year	Pandemic
Europe	26.77	0.0336	−0.1530
Northern Europe	−252.76	0.1724 ***	−2.2187 ***
Southern Europe	−84.61	0.0896 *	−2.6746 ***
Eastern Europe	−65.71	0.0791	0.5507
Western Europe	59.61	0.0179	−1.8149 *

* *p* < 0.05; *** *p* < 0.001.

**Table 5:** Segmented regression of DTP 3 coverage in European countries.

Nation	Intercept	Year	Pandemic	Interaction
Albania	−31.2673	0.0645	−1.6521 **	N/A
Andorra	−252.7788	0.1742	−0.8535	N/A−
Austria	−677.9055	0.3806	−6.8187	N/A−
Belarus	−23.6828	0.0602	−0.2919	N/A−
Belgium	145.8932	−0.0237	−0.3228	N/A−
Croatia	687.9301	−0.2946 ***	0.4016	N/A−
Cyprus	270.5469	−0.0860	−1.6544 ^†^	N/A−
Czechia	569.6045	−0.2344 ***	−0.9975	N/A−
Denmark	−1253.2089	0.6688 ***	−1.8051	N/A−
Estonia	634.5661	−0.2688 ***	−0.9021 *	N/A−
Finland	1455.1928	−0.6753 ***	0.1131	N/A−
France	643.1797	−0.2710 ***	0.5816	N/A
Germany	1058.9201	−0.4796 ***	1.5304 *	N/A−
Greece	42.6651	0.0280 ^†^	−0.1802	N/A−
Hungary	99.0000	0.0000 ***	0.0000	N/A−
Iceland	1317.9547	−0.6086 ***	4.5846 **	N/A−
Ireland	−247.6943	0.1699	−3.2433 *	−1.8087 **
Italy	355.2488	−0.1290 *	0.0899	N/A
Latvia	−494.0307	0.2925 ^†^	−0.7037	N/A−
Lithuania	591.5722	−0.2473 **	−1.3813	N/A−
Luxembourg	99.0000	0.0000	0.0000	N/A−
Malta	−3965.2956	2.0150 ***	−9.8854	N/A−
Monaco	99.0000	0.0000	0.0000	N/A−
Montenegro	1272.4608	−0.5871 **	−3.6447 ^†^	N/A−
Netherlands (Kingdom of the)	667.1475	−0.2839 ***	0.1977	N/A−
North Macedonia	527.0100	−0.2151 ***	−8.5288 ***	N/A−
Norway	−368.5200	0.2301 ***	0.1076	N/A−
Poland	695.2581	−0.2968 ***	−1.3290 ^†^	N/A−
Portugal	−222.5545	0.1591 ***	−0.1465	N/A
Republic of Moldova	432.4086	−0.1699	−1.9710	N/A−
Romania	1960.9631	−0.9290 ***	1.0756	N/A−
Russian Federation	270.4040	−0.0860 ***	0.4885 *	N/A−
San Marino	789.8433	−0.3484*	4.9212 *	N/A−
Serbia	179.9877	−0.0430	−0.0415	N/A−
Slovakia	669.0760	−0.2839 ***	1.5191 *	N/A−
Slovenia	679.7028	−0.2903 *	−3.0657 *	N/A−
Spain	321.4823	−0.1118 *	−2.4221 **	N/A−
Sweden	253.5922	−0.0774 ^†^	−1.3389 ^†^	−1.2494 ***
Switzerland	−190.1452	0.1419 ***	−1.0274 **	−0.4516 *
Türkiye	−587.3172	0.3398 **	−1.8081	N/A
Ukraine	6563.0914	−3.2301 *	45.3210 *	N/A−
United Kingdom	−170.2197	0.1312^†^	−2.4664 **	N/A−

N/A = No interaction ^†^ *p* < 0.10 * *p* < 0.05 ** *p* < 0.01 *** *p* < 0.001.

**Table 6:** Comparison of the findings of segmented regression, comparison of rates 2019–2023, and joinpoint.

Country	Segmented	Chi-Square	Joinpoint
Albania	−	−	−
Austria	−	−	
Belgium		+	+
Bosnia			+
Bulgaria		−	
Croatia		−	
Cyprus	−		
Czechia		−	
Estonia	−	−	
Ireland	−	−	−
Italy		−	
Latvia			
Lithuania	−	−	
Moldavia	−	−	
Montenegro	−	−	
Netherland		−	
North Macedonia	−	−	
Norway		−	
Poland	−	−	−
Romania		−	
Serbia		−	
Slovakia		−	−
Slovenia	−	−	
Spain	−	−	
Sweden	−	−	−
Switzerland	−	−	−
Türkiye	−		
Ukraine		+	
United Kingdom	−	−	

+ Green increase; − pink decrease.

## Data Availability

Data were obtained from the following resources available in the public domain: World Bank, United Nations, and UNICEF. https://data.worldbank.org/indicator/SP.POP.TOTL (accessed on 23 October 2024). https://data.unicef.org/resources/data_explorer/unicef_f/?ag=UNICEF&df=GLOBAL_DATAFLOW&ver=1.0&dq=.DM_BRTS..&startPeriod=2019&endPeriod=2023, https://population.un.org/wpp (accessed on 23 October 2024).

## Supplementary Materials

The following supporting information can be downloaded at: https://www.mdpi.com/article/10.3390/vaccines13010006/s1, Table S1: Confidence Intervals of Joinpoints for European Regions Whose 95% Confidence Intervals Included the COVID-19 Pandemic Year (2020); Table S2: Confidence Intervals of Joinpoints for European Countries Whose 95% Confidence Intervals Included the COVID-19 Pandemic Year (2020).
