# Supplementary material for: The Impact of COVID-19 on DTP3 Vaccination Coverage in Europe (2012–2023)
Source: Vaccines (Basel). 2024 Dec 24;13(1):6. doi: 10.3390/vaccines13010006 (PMC11768563; doi:10.3390/vaccines13010006)
Supplement: Supplementary file 1 [file vaccines-13-00006-s001.zip › vaccines-3347622-supplementary.pdf]

Supplementary Materials

Table S1. Confidence Intervals of Joinpoints for European Regions Whose 95% Confidence Intervals Included the COVID-19 Pandemic Year (2020).

Region	Estimate	Lower 95% CI	Upper 95%CI
South	**2021**	2021	2021
West	**2020**	2015	2021

Table S2. Confidence Intervals of Joinpoints for European Countries Whose 95% Confidence Intervals Included the COVID-19 Pandemic Year (2020).

Country	Estimate	Lower 95% CI	Upper 95%CI
**Albania**	2019	2015	2021
**Belgium**	**2019**	2017	2021
**Bosnia**	**2021**	2020	2021
**Ireland**	**2021**	2019	2021
**Poland**	**2019**	2019	2020
**Slovakia**	**2021**	2019	2021
**Sweden**	**2021**	2020	2021
**Switzerland**	**2021**	2016	2021
